# 8,13,26-Trioxa-23-thia-21-aza­penta­cyclo­[18.6.0.0^2,7^.0^14,19^.0^21,25^]hexa­cosa-2(7),3,5,14,16,18-hexa­ene

**DOI:** 10.1107/S1600536813012804

**Published:** 2013-05-15

**Authors:** Seenivasan Karthiga Devi, Thothadri Srinivasan, Santhanagopalan Purushothaman, Raghavachary Raghunathan, Devadasan Velmurugan

**Affiliations:** a Centre of Advanced Study in Crystallography and Biophysics, University of Madras, Guindy Campus, Chennai 600 025, India; b Department of Organic Chemistry, University of Madras, Guindy Campus, Chennai 600 025, India

## Abstract

In the title compound, C_21_H_23_NO_3_S, both the thia­zole and oxazolidine rings adopt twist conformations. The mean plane of the thia­zole ring makes a dihedral angle of 61.02 (7)° with the oxazolidine ring mean plane, and dihedral angles of 22.72 (6) and 75.07 (6)° with the benzene rings. The benzene rings are almost perpendicular to one another, making a dihedral angle of 89.14 (6)°. There are bifurcated intra­molecular C—H⋯O hydrogen bonds in the mol­ecular structure. In the crystal, mol­ecules are linked *via* C—H⋯π inter­actions, forming chains propagating along [100].

## Related literature

For the biological activity of thia­zole derivatives, see: Guo *et al.* (2006 ▶); Karegoudar *et al.* (2008 ▶); Reddy *et al.* (1999 ▶).
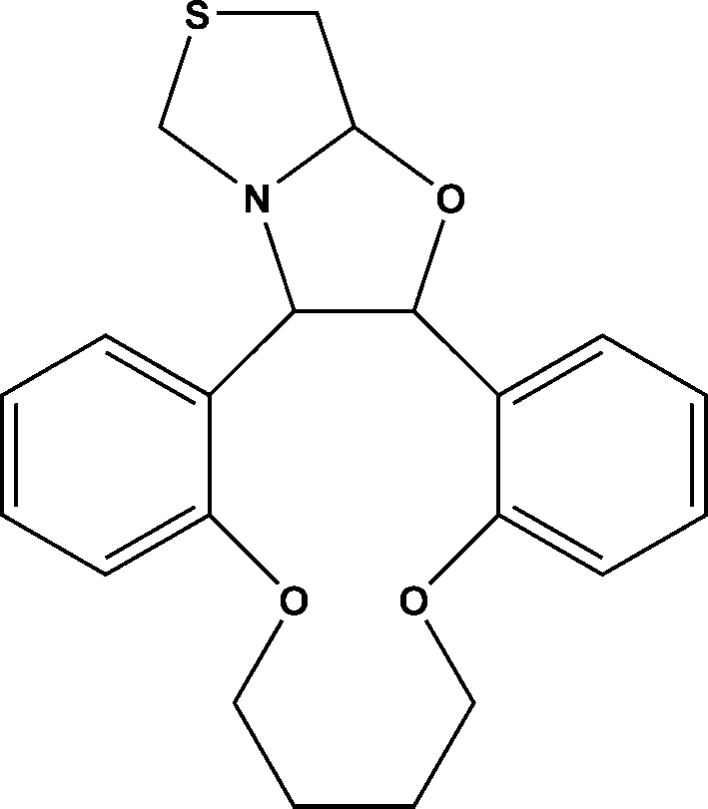


## Experimental

### 

#### Crystal data

C_21_H_23_NO_3_S
*M* *_r_* = 369.46
Monoclinic, 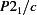

*a* = 9.9858 (5) Å
*b* = 17.8830 (8) Å
*c* = 10.2054 (4) Å
β = 94.663 (2)°
*V* = 1816.41 (14) Å^3^
*Z* = 4
Mo *K*α radiation
μ = 0.20 mm^−1^
*T* = 293 K
0.30 × 0.25 × 0.20 mm

#### Data collection

Bruker SMART APEXII area-detector diffractometer
Absorption correction: multi-scan (*SADABS*; Bruker, 2008 ▶) *T*_min_ = 0.943, *T*_max_ = 0.961
29183 measured reflections
7768 independent reflections
5150 reflections with *I* > 2σ(*I*)
*R*_int_ = 0.034

#### Refinement

*R*[*F* ^2^ > 2σ(*F* ^2^)] = 0.047
*wR*(*F* ^2^) = 0.152
*S* = 1.02
7768 reflections
235 parameters
H-atom parameters constrained
Δρ_max_ = 0.38 e Å^−3^
Δρ_min_ = −0.23 e Å^−3^

### 

Data collection: *APEX2* (Bruker, 2008 ▶); cell refinement: *SAINT* (Bruker, 2008 ▶); data reduction: *SAINT*; program(s) used to solve structure: *SHELXS97* (Sheldrick, 2008 ▶); program(s) used to refine structure: *SHELXL97* (Sheldrick, 2008 ▶); molecular graphics: *ORTEP-3 for Windows* (Farrugia, 2012 ▶); software used to prepare material for publication: *SHELXL97* and *PLATON* (Spek, 2009 ▶).

## Supplementary Material

Crystal structure: contains datablock(s) global, I. DOI: 10.1107/S1600536813012804/su2596sup1.cif

Structure factors: contains datablock(s) I. DOI: 10.1107/S1600536813012804/su2596Isup2.hkl

Supplementary material file. DOI: 10.1107/S1600536813012804/su2596Isup3.cml

Additional supplementary materials:  crystallographic information; 3D view; checkCIF report

## Figures and Tables

**Table 1:** Hydrogen-bond geometry (Å, °) *Cg*1 is the centroid of the C9–C14 ring.

*D*—H⋯*A*	*D*—H	H⋯*A*	*D*⋯*A*	*D*—H⋯*A*
C8—H8⋯O1	0.98	2.30	2.9593 (13)	123
C8—H8⋯O2	0.98	2.30	2.7146 (14)	104
C3—H3⋯*Cg*1^i^	0.93	2.99	3.8672 (14)	158
C16—H16*B*⋯*Cg*1^ii^	0.97	2.81	3.7400 (17)	160

Symmetry codes: (i) 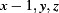; (ii) 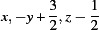.

**Fig. 1.**
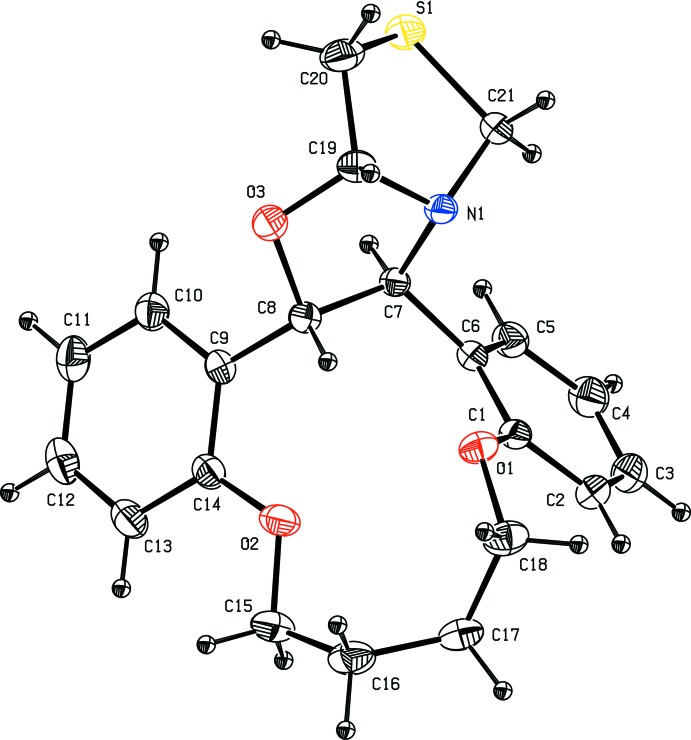
The molecular structure of the title molecule, with atom labelling. Displacement ellipsoids are drawn at the 30% probability level.

## supplementary crystallographic information

### Comment

Thiazole derivatives have a variety of physiological effects, such as antiinflammatory (Guo *et al.*, 2006) and antimicrobial (Karegoudar *et al.*, 2008). Against this background, we report herein on the crystal structure of the title thiazole derivative.

In the title compound, Fig. 1, both the thiazole ring (S1/N1/C19-C21) and the oxazolidine ring (O3/N1/C7/C8/C19) adopt *twist* conformations. The former is twisted on bond S1-C21, while the latter is twisted on bond C8-C7. Their mean planes are inclined to one another by 61.02 (7)°. The mean plane of the thiazole ring makes a dihedral angle of 22.72 (6)° with benzene ring (C1-C6), and a dihedral angle of 75.07 (6)° with the other benzene ring (C9-C14). The dihedral angle between the two benzene rings is 89.14 (6)°. The oxazolidine ring mean plane makes dihedral angles of 81.69 (6)° with benzene ring (C1-C6) and 68.92 (6)° with benzene ring (C9-C14).

The molecule features bifurcated intramolecular C–H···O hydrogen bonds (Table 1).

In the crystal, molecules are linked via C-H···π interactions forming chains along direction [100]; see Table 1.

### Experimental

A mixture of 2,2'-(butane-1,4-diylbis(oxy))dibenzaldehyde (1 mMol) and thiazolidine-4-carboxylic acid (1 mMol) was refluxed in acetonitrile (30 ml) for about 6 hrs under N_2_ atm. After the completion of reaction as indicated by TLC, acetonitrile was evaporated under reduced pressure. The crude product was purified by column chromatography using hexane: EtOAc (8:2) mixture as eluent. Block-like colourless crystals of the title compound, suitable for X-ray diffraction, were obtained by slow evaporation of a solution in ethyl acetate at room temperature.

### Refinement

The H atoms were placed in calculated positions and treated as riding atoms: C—H = 0.93 - 0.98 Å, with U_iso_(H) = 1.5U_eq_(C) for methyl H atoms and = 1.2U_eq_(C) for other H atoms.

### Crystal data

**Table:**

C_21_H_23_NO_3_S	*F*(000) = 784
*M**_r_* = 369.46	*D*_x_ = 1.351 Mg m^−^^3^
Monoclinic, *P*2_1_/*c*	Mo *K*α radiation, λ = 0.71073 Å
Hall symbol: -P 2ybc	Cell parameters from 7768 reflections
*a* = 9.9858 (5) Å	θ = 2.1–34.8°
*b* = 17.8830 (8) Å	µ = 0.20 mm^−^^1^
*c* = 10.2054 (4) Å	*T* = 293 K
β = 94.663 (2)°	Block, colourless
*V* = 1816.41 (14) Å^3^	0.30 × 0.25 × 0.20 mm
*Z* = 4	

### Data collection

**Table:**

Bruker SMART APEXII area-detector diffractometer	7768 independent reflections
Radiation source: fine-focus sealed tube	5150 reflections with *I* > 2σ(*I*)
Graphite monochromator	*R*_int_ = 0.034
ω and φ scans	θ_max_ = 34.8°, θ_min_ = 2.1°
Absorption correction: multi-scan (*SADABS*; Bruker, 2008)	*h* = −13→15
*T*_min_ = 0.943, *T*_max_ = 0.961	*k* = −28→28
29183 measured reflections	*l* = −15→16

### Refinement

**Table:**

Refinement on *F*^2^	Primary atom site location: structure-invariant direct methods
Least-squares matrix: full	Secondary atom site location: difference Fourier map
*R*[*F*^2^ > 2σ(*F*^2^)] = 0.047	Hydrogen site location: inferred from neighbouring sites
*wR*(*F*^2^) = 0.152	H-atom parameters constrained
*S* = 1.02	*w* = 1/[σ^2^(*F*_o_^2^) + (0.0779*P*)^2^ + 0.2141*P*] where *P* = (*F*_o_^2^ + 2*F*_c_^2^)/3
7768 reflections	(Δ/σ)_max_ = 0.002
235 parameters	Δρ_max_ = 0.38 e Å^−^^3^
0 restraints	Δρ_min_ = −0.23 e Å^−^^3^

### Special details

**Table:**

Geometry. Bond distances, angles etc. have been calculated using the rounded fractional coordinates. All su's are estimated from the variances of the (full) variance-covariance matrix. The cell esds are taken into account in the estimation of distances, angles and torsion angles
Refinement. Refinement of F^2^ against ALL reflections. The weighted R-factor wR and goodness of fit S are based on F^2^, conventional R-factors R are based on F, with F set to zero for negative F^2^. The threshold expression of F^2^ > 2sigma(F^2^) is used only for calculating R-factors(gt) etc. and is not relevant to the choice of reflections for refinement. R-factors based on F^2^ are statistically about twice as large as those based on F, and R- factors based on ALL data will be even larger.

### Fractional atomic coordinates and isotropic or equivalent isotropic displacement parameters (Å^2^)

**Table:**

	*x*	*y*	*z*	*U*_iso_*/*U*_eq_	
S1	0.74184 (4)	1.12013 (2)	0.65807 (4)	0.0494 (1)	
O1	0.57658 (8)	0.80765 (4)	0.59355 (7)	0.0381 (2)	
O2	0.78980 (10)	0.74731 (5)	0.76920 (9)	0.0485 (3)	
O3	0.89870 (8)	0.94723 (5)	0.64326 (9)	0.0436 (3)	
N1	0.67629 (9)	0.98089 (5)	0.59254 (9)	0.0340 (2)	
C1	0.50176 (11)	0.84125 (6)	0.68238 (10)	0.0325 (3)	
C2	0.37387 (12)	0.81698 (7)	0.70780 (13)	0.0429 (3)	
C3	0.30109 (13)	0.85474 (8)	0.79647 (15)	0.0506 (4)	
C4	0.35388 (13)	0.91678 (8)	0.86141 (14)	0.0500 (4)	
C5	0.48200 (12)	0.94056 (7)	0.83674 (12)	0.0398 (3)	
C6	0.55823 (10)	0.90457 (6)	0.74765 (10)	0.0305 (2)	
C7	0.68950 (10)	0.94036 (5)	0.71960 (9)	0.0289 (2)	
C8	0.81406 (10)	0.89322 (6)	0.70168 (10)	0.0322 (3)	
C9	0.88694 (10)	0.86250 (6)	0.82490 (11)	0.0337 (3)	
C10	0.96886 (12)	0.90696 (7)	0.90900 (12)	0.0431 (3)	
C11	1.03721 (13)	0.87723 (9)	1.02151 (14)	0.0512 (4)	
C12	1.02422 (13)	0.80285 (9)	1.04927 (13)	0.0512 (4)	
C13	0.94301 (13)	0.75665 (8)	0.96711 (13)	0.0465 (4)	
C14	0.87363 (11)	0.78676 (6)	0.85559 (11)	0.0372 (3)	
C15	0.77313 (15)	0.66932 (7)	0.78900 (14)	0.0506 (4)	
C16	0.69047 (16)	0.63866 (7)	0.67137 (15)	0.0525 (4)	
C17	0.54971 (14)	0.67230 (7)	0.64497 (14)	0.0476 (4)	
C18	0.53496 (15)	0.73498 (7)	0.54430 (12)	0.0475 (4)	
C19	0.81413 (12)	0.99003 (6)	0.55430 (11)	0.0385 (3)	
C20	0.85491 (15)	1.07215 (8)	0.55857 (18)	0.0572 (5)	
C21	0.61277 (12)	1.05348 (6)	0.59768 (13)	0.0427 (3)	
H2	0.33710	0.77500	0.66470	0.0510*	
H3	0.21570	0.83800	0.81240	0.0610*	
H4	0.30480	0.94240	0.92070	0.0600*	
H5	0.51810	0.98210	0.88160	0.0480*	
H7	0.71300	0.97700	0.78920	0.0350*	
H8	0.79150	0.85250	0.63950	0.0390*	
H10	0.97830	0.95740	0.89000	0.0520*	
H11	1.09130	0.90770	1.07740	0.0610*	
H12	1.07030	0.78290	1.12410	0.0610*	
H13	0.93510	0.70610	0.98650	0.0560*	
H15A	0.72790	0.66060	0.86820	0.0610*	
H15B	0.86000	0.64480	0.79860	0.0610*	
H16A	0.68140	0.58510	0.68280	0.0630*	
H16B	0.73940	0.64660	0.59420	0.0630*	
H17A	0.48840	0.63240	0.61600	0.0570*	
H17B	0.52130	0.69110	0.72750	0.0570*	
H18A	0.58740	0.72240	0.47140	0.0570*	
H18B	0.44150	0.73790	0.51030	0.0570*	
H19	0.81950	0.97070	0.46500	0.0460*	
H20A	0.84880	1.09290	0.47050	0.0690*	
H20B	0.94670	1.07740	0.59640	0.0690*	
H21A	0.54100	1.05200	0.65630	0.0510*	
H21B	0.57490	1.06800	0.51090	0.0510*	

### Atomic displacement parameters (Å^2^)

**Table:**

	*U*^11^	*U*^22^	*U*^33^	*U*^12^	*U*^13^	*U*^23^
S1	0.0521 (2)	0.0315 (2)	0.0628 (2)	−0.0031 (1)	−0.0075 (2)	−0.0057 (1)
O1	0.0453 (4)	0.0319 (4)	0.0373 (4)	−0.0076 (3)	0.0047 (3)	−0.0043 (3)
O2	0.0605 (6)	0.0298 (4)	0.0528 (5)	−0.0035 (3)	−0.0103 (4)	0.0067 (3)
O3	0.0342 (4)	0.0459 (5)	0.0514 (5)	−0.0017 (3)	0.0071 (3)	0.0131 (4)
N1	0.0372 (4)	0.0292 (4)	0.0343 (4)	−0.0051 (3)	−0.0041 (3)	0.0056 (3)
C1	0.0343 (5)	0.0290 (4)	0.0336 (5)	−0.0023 (4)	−0.0007 (4)	0.0051 (4)
C2	0.0359 (5)	0.0394 (6)	0.0527 (7)	−0.0063 (4)	−0.0007 (5)	0.0065 (5)
C3	0.0338 (6)	0.0527 (7)	0.0663 (8)	0.0000 (5)	0.0097 (5)	0.0116 (6)
C4	0.0426 (6)	0.0506 (7)	0.0590 (8)	0.0076 (5)	0.0172 (5)	0.0041 (6)
C5	0.0399 (5)	0.0364 (5)	0.0435 (6)	0.0037 (4)	0.0058 (4)	−0.0010 (4)
C6	0.0309 (4)	0.0277 (4)	0.0323 (4)	0.0010 (3)	−0.0006 (3)	0.0042 (3)
C7	0.0315 (4)	0.0247 (4)	0.0299 (4)	−0.0011 (3)	−0.0016 (3)	0.0012 (3)
C8	0.0319 (4)	0.0299 (4)	0.0347 (5)	0.0002 (3)	0.0031 (3)	0.0018 (4)
C9	0.0298 (4)	0.0331 (5)	0.0380 (5)	0.0053 (4)	0.0017 (4)	0.0011 (4)
C10	0.0387 (6)	0.0412 (6)	0.0479 (6)	0.0014 (4)	−0.0049 (4)	−0.0024 (5)
C11	0.0407 (6)	0.0623 (9)	0.0484 (7)	0.0022 (5)	−0.0094 (5)	−0.0050 (6)
C12	0.0423 (6)	0.0658 (9)	0.0443 (6)	0.0132 (6)	−0.0043 (5)	0.0077 (6)
C13	0.0456 (6)	0.0459 (7)	0.0477 (6)	0.0105 (5)	0.0015 (5)	0.0108 (5)
C14	0.0360 (5)	0.0349 (5)	0.0405 (5)	0.0060 (4)	0.0016 (4)	0.0024 (4)
C15	0.0603 (8)	0.0290 (5)	0.0624 (8)	0.0007 (5)	0.0048 (6)	0.0076 (5)
C16	0.0625 (8)	0.0299 (5)	0.0666 (9)	−0.0033 (5)	0.0146 (6)	−0.0068 (5)
C17	0.0575 (7)	0.0320 (5)	0.0539 (7)	−0.0119 (5)	0.0087 (6)	−0.0045 (5)
C18	0.0639 (8)	0.0369 (6)	0.0414 (6)	−0.0127 (5)	0.0025 (5)	−0.0084 (5)
C19	0.0454 (6)	0.0340 (5)	0.0367 (5)	−0.0031 (4)	0.0075 (4)	0.0031 (4)
C20	0.0490 (7)	0.0384 (6)	0.0860 (11)	−0.0081 (5)	0.0170 (7)	0.0104 (7)
C21	0.0378 (5)	0.0338 (5)	0.0543 (7)	−0.0020 (4)	−0.0087 (5)	0.0119 (5)

### Geometric parameters (Å, º)

**Table:**

S1—C20	1.7968 (16)	C16—C17	1.533 (2)
S1—C21	1.8255 (12)	C17—C18	1.5196 (18)
O1—C1	1.3606 (13)	C19—C20	1.5236 (18)
O1—C18	1.4423 (15)	C2—H2	0.9300
O2—C14	1.3623 (14)	C3—H3	0.9300
O2—C15	1.4210 (15)	C4—H4	0.9300
O3—C8	1.4438 (14)	C5—H5	0.9300
O3—C19	1.4137 (14)	C7—H7	0.9800
N1—C7	1.4820 (13)	C8—H8	0.9800
N1—C19	1.4700 (15)	C10—H10	0.9300
N1—C21	1.4476 (14)	C11—H11	0.9300
C1—C2	1.3930 (16)	C12—H12	0.9300
C1—C6	1.4081 (15)	C13—H13	0.9300
C2—C3	1.3820 (19)	C15—H15A	0.9700
C3—C4	1.375 (2)	C15—H15B	0.9700
C4—C5	1.3905 (18)	C16—H16A	0.9700
C5—C6	1.3903 (16)	C16—H16B	0.9700
C6—C7	1.5070 (14)	C17—H17A	0.9700
C7—C8	1.5259 (14)	C17—H17B	0.9700
C8—C9	1.5047 (15)	C18—H18A	0.9700
C9—C10	1.3870 (16)	C18—H18B	0.9700
C9—C14	1.3990 (15)	C19—H19	0.9800
C10—C11	1.3927 (19)	C20—H20A	0.9700
C11—C12	1.368 (2)	C20—H20B	0.9700
C12—C13	1.390 (2)	C21—H21A	0.9700
C13—C14	1.3918 (17)	C21—H21B	0.9700
C15—C16	1.504 (2)		
			
C20—S1—C21	87.50 (6)	C6—C5—H5	119.00
C1—O1—C18	118.15 (9)	N1—C7—H7	108.00
C14—O2—C15	119.29 (10)	C6—C7—H7	108.00
C8—O3—C19	106.75 (8)	C8—C7—H7	108.00
C7—N1—C19	105.55 (8)	O3—C8—H8	110.00
C7—N1—C21	114.52 (9)	C7—C8—H8	110.00
C19—N1—C21	109.41 (9)	C9—C8—H8	110.00
O1—C1—C2	123.22 (10)	C9—C10—H10	119.00
O1—C1—C6	116.73 (9)	C11—C10—H10	119.00
C2—C1—C6	120.03 (10)	C10—C11—H11	120.00
C1—C2—C3	120.57 (12)	C12—C11—H11	120.00
C2—C3—C4	120.55 (12)	C11—C12—H12	120.00
C3—C4—C5	118.80 (12)	C13—C12—H12	120.00
C4—C5—C6	122.52 (12)	C12—C13—H13	120.00
C1—C6—C5	117.53 (10)	C14—C13—H13	120.00
C1—C6—C7	124.94 (9)	O2—C15—H15A	110.00
C5—C6—C7	117.30 (9)	O2—C15—H15B	110.00
N1—C7—C6	110.92 (8)	C16—C15—H15A	110.00
N1—C7—C8	100.42 (8)	C16—C15—H15B	110.00
C6—C7—C8	121.22 (8)	H15A—C15—H15B	108.00
O3—C8—C7	100.96 (8)	C15—C16—H16A	108.00
O3—C8—C9	109.25 (8)	C15—C16—H16B	108.00
C7—C8—C9	116.43 (8)	C17—C16—H16A	108.00
C8—C9—C10	121.92 (10)	C17—C16—H16B	108.00
C8—C9—C14	119.49 (10)	H16A—C16—H16B	107.00
C10—C9—C14	118.58 (10)	C16—C17—H17A	108.00
C9—C10—C11	121.08 (12)	C16—C17—H17B	108.00
C10—C11—C12	119.57 (13)	C18—C17—H17A	108.00
C11—C12—C13	120.88 (13)	C18—C17—H17B	108.00
C12—C13—C14	119.37 (13)	H17A—C17—H17B	107.00
O2—C14—C9	114.90 (10)	O1—C18—H18A	109.00
O2—C14—C13	124.59 (11)	O1—C18—H18B	109.00
C9—C14—C13	120.51 (11)	C17—C18—H18A	109.00
O2—C15—C16	107.90 (11)	C17—C18—H18B	109.00
C15—C16—C17	115.67 (12)	H18A—C18—H18B	108.00
C16—C17—C18	116.46 (12)	O3—C19—H19	109.00
O1—C18—C17	114.82 (10)	N1—C19—H19	109.00
O3—C19—N1	107.02 (9)	C20—C19—H19	109.00
O3—C19—C20	111.05 (10)	S1—C20—H20A	110.00
N1—C19—C20	110.70 (10)	S1—C20—H20B	110.00
S1—C20—C19	107.32 (10)	C19—C20—H20A	110.00
S1—C21—N1	107.36 (8)	C19—C20—H20B	110.00
C1—C2—H2	120.00	H20A—C20—H20B	109.00
C3—C2—H2	120.00	S1—C21—H21A	110.00
C2—C3—H3	120.00	S1—C21—H21B	110.00
C4—C3—H3	120.00	N1—C21—H21A	110.00
C3—C4—H4	121.00	N1—C21—H21B	110.00
C5—C4—H4	121.00	H21A—C21—H21B	109.00
C4—C5—H5	119.00		
			
C21—S1—C20—C19	−32.36 (10)	C4—C5—C6—C7	173.95 (11)
C20—S1—C21—N1	39.77 (9)	C4—C5—C6—C1	−0.75 (17)
C18—O1—C1—C2	−13.60 (15)	C5—C6—C7—C8	141.10 (10)
C18—O1—C1—C6	167.96 (10)	C5—C6—C7—N1	−101.65 (11)
C1—O1—C18—C17	−68.41 (14)	C1—C6—C7—C8	−44.64 (14)
C14—O2—C15—C16	−172.86 (11)	C1—C6—C7—N1	72.61 (12)
C15—O2—C14—C9	177.78 (11)	N1—C7—C8—C9	160.85 (9)
C15—O2—C14—C13	−2.20 (18)	C6—C7—C8—O3	165.12 (8)
C8—O3—C19—C20	140.90 (10)	C6—C7—C8—C9	−76.76 (12)
C8—O3—C19—N1	19.98 (11)	N1—C7—C8—O3	42.72 (9)
C19—O3—C8—C9	−162.56 (9)	C7—C8—C9—C10	−76.25 (13)
C19—O3—C8—C7	−39.33 (10)	O3—C8—C9—C14	−141.97 (10)
C21—N1—C19—C20	11.04 (14)	C7—C8—C9—C14	104.54 (11)
C19—N1—C7—C6	−160.77 (8)	O3—C8—C9—C10	37.25 (14)
C7—N1—C19—C20	−112.65 (11)	C8—C9—C14—C13	178.22 (10)
C19—N1—C21—S1	−35.51 (11)	C8—C9—C10—C11	−178.96 (11)
C21—N1—C7—C8	−151.80 (9)	C8—C9—C14—O2	−1.76 (15)
C21—N1—C19—O3	132.18 (9)	C14—C9—C10—C11	0.26 (17)
C19—N1—C7—C8	−31.39 (9)	C10—C9—C14—C13	−1.02 (17)
C7—N1—C19—O3	8.49 (10)	C10—C9—C14—O2	179.01 (10)
C21—N1—C7—C6	78.83 (11)	C9—C10—C11—C12	0.45 (19)
C7—N1—C21—S1	82.72 (10)	C10—C11—C12—C13	−0.4 (2)
C2—C1—C6—C7	−174.06 (10)	C11—C12—C13—C14	−0.3 (2)
C2—C1—C6—C5	0.19 (16)	C12—C13—C14—O2	−178.97 (12)
C6—C1—C2—C3	0.27 (18)	C12—C13—C14—C9	1.06 (18)
O1—C1—C6—C5	178.68 (10)	O2—C15—C16—C17	−60.14 (15)
O1—C1—C2—C3	−178.11 (11)	C15—C16—C17—C18	96.72 (15)
O1—C1—C6—C7	4.43 (15)	C16—C17—C18—O1	−80.88 (15)
C1—C2—C3—C4	−0.2 (2)	O3—C19—C20—S1	−100.26 (11)
C2—C3—C4—C5	−0.3 (2)	N1—C19—C20—S1	18.46 (13)
C3—C4—C5—C6	0.8 (2)		

### Hydrogen-bond geometry (Å, º)

Cg1 is the centroid of the C9–C14 ring.

**Table:**

*D*—H···*A*	*D*—H	H···*A*	*D*···*A*	*D*—H···*A*
C8—H8···O1	0.98	2.30	2.9593 (13)	123
C8—H8···O2	0.98	2.30	2.7146 (14)	104
C3—H3···*Cg*1^i^	0.93	2.99	3.8672 (14)	158
C16—H16*B*···*Cg*1^ii^	0.97	2.81	3.7400 (17)	160

Symmetry codes: (i) *x*−1, *y*, *z*; (ii) *x*, −*y*+3/2, *z*−1/2.
